# Management of Concurrent Bipolar I Disorder and Compulsive Sexual Behavior Disorder: A Case Report

**DOI:** 10.7759/cureus.45016

**Published:** 2023-09-11

**Authors:** Jaclyn Fiandor-Montesino, Melanie Kusakavitch, Raju Kakarlapudi, Manoj Puthiyathu

**Affiliations:** 1 Medicine, School of Medicine, St. George's University, Hackensack, USA; 2 Medicine, College of Osteopathic Medicine, University of New England, Portland, USA; 3 Psychiatry, Bergen New Bridge Medical Center, Paramus, USA

**Keywords:** compulsive sexual behavior disorder, naltrexone, bipolar i disorder, pornography, sexual behavior

## Abstract

Pornography addiction is not currently recognized in the Diagnostic and Statistical Manual of Mental Disorders Fifth Edition (DSM-V), but it can be broadly classified under compulsive sexual behavior disorder (CSBD) in the International Classification for Diseases (ICD). An increased incidence of CSBD has been reported in patients with bipolar I disorder (BPI) as an independent compulsion, not secondary to mania. Due to the ambiguity of identifying pornography addiction in the DSM-V, approved treatment for patients with a concurrent diagnosis of CSBD in the context of BPI is limited. We report the case of a patient with CSBD with concurrent BPI and investigate the treatment strategy taken in an inpatient setting. Though there are no FDA-approved treatment strategies currently approved for patients with concurrent CSBD and BPI, selective serotonin reuptake inhibitors, mood stabilizers, naltrexone, and cognitive behavioral therapy can be used in combination for maximal benefit.

## Introduction

The Diagnostic and Statistical Manual of Mental Disorders Fifth Edition (DSM-V) does not provide a formal definition for “pornography addiction” [1]. Pornography addiction has been categorized under compulsive sexual behavior disorder (CSBD) in the International Classification for Diseases (ICD) in order to help classify and treat patients [1]. Individuals who do not experience impairment of function but still exhibit a high sex drive do not meet the criteria for this disorder [1]. This classification assisted in differentiating CSBD from other health conditions in this patient.

In this case, pornography addiction in the setting of bipolar I disorder (BPI) has precipitated severe impairment in the function and integration of the patient. Patients with BPI have an increased incidence of CSBD [2]. The sexual behaviors must be persistent and occur independently of manic episodes to provide a basis for diagnosis [2]. In regard to this patient, his sexual compulsions occur outside episodes of mania and negatively impact his occupation, relationships, physical health, mental health, and overall functioning. The purpose of this case report is to review current treatment for CSBD including psychotherapy and pharmacological courses.

## Case presentation

A 31-year-old male with a past psychiatric history of BPI, cannabis use disorder, and panic attacks with no previous psychiatry-related hospitalizations or significant medical history presented to the emergency department with worsening suicidal ideation for several days. The patient had been experiencing worsening depressive symptomatology including depressed mood, anhedonia, hopelessness, guilt, and decreased sleep. There was no current manic or psychotic symptomatology. The patient reported that he is currently under the care of a psychiatrist and is prescribed quetiapine 50 milligrams (mg) once daily per os (PO), which he is adherent to. On mental status examination, the patient appeared well groomed and was in adequate behavioral control. His mood was “depressed” with a mood-congruent affect. The patient had a linear thought process with no current homicidal ideations or suicidal ideations at the time of interview, visual or auditory hallucinations, or delusions. There was no overt evidence of perceptual disturbances. The patient’s insight and judgment were fair. Lab workup including complete blood count and a comprehensive metabolic panel was normal (Table 1). Urine toxicology was significant for cannabinoids (Table 2). The patient reported using marijuana multiple times weekly for the past 15 years and drinking one pint of whiskey on the weekends with no history of withdrawal symptoms. The patient was admitted to the dual diagnosis inpatient unit and was seen by the psychiatry team.

**Table 1:** Initial laboratory results

Serum	Results	Reference range
White blood cells (x10^3^/uL)	5.3	4.5–11.0
Hemoglobin (g/dL)	14.4	12.0–16.0
Mean corpuscular volume (fL)	85.2	80.0–100.0
Platelet count (x10^3^/uL)	196	140–450
Glucose (mg/dL)	68	136–145
Blood urea nitrogen (mg/dL)	15	5–25
Creatinine (mg/dL)	1.05	0.44–1.0
Sodium (mmol/L)	140	135–146
Potassium (mmol/L)	3.7	3.5–5.2
Chloride (mmol/L)	104	96–110
Calcium (mg/dL)	9.2	8.5–10.5
Magnesium (mg/dL)	2.0	1.3–2.5
Bicarbonate (mmol/L)	29.2	24–31
Alkaline phosphatase (U/L)	73	38–126
Albumin (g/dL)	4.3	3.5–5
Bilirubin, total (mg/dL)	0.8	0.2–1.3
Aspartate aminotransferase (U/L)	15	10–42
Alanine aminotransferase (U/L)	7	7–52

**Table 2:** Urine toxicology results

Serum	Results
Methadone	Negative
Opiates	Negative
Barbiturates	Negative
Amphetamines	Negative
Cannabinoids	Positive
Cocaine	Negative
Benzodiazepine	Negative
Phencyclidine	Negative

During the initial evaluation by the inpatient psychiatry team, the patient stated that his recent stressors leading to his depressive episode included worsening self-reported “pornography addiction.” The patient revealed that in his early teenage years, he began watching internet pornography as a coping mechanism secondary to social anxiety and bullying in school. He stated that at 16 years of age, watching pornography and masturbating began interfering with his life at school, relationships with family members, and daily activities. The inability to masturbate caused him a great deal of distress. He stated that over the next few years, he began watching pornography and masturbating up to 18 times per day and reported that there were days he masturbated 40 times to the point of injuring his genitals. He reported his longest period sustained from watching pornography and masturbating was over a 34-day period, during which he sought support via an internet forum, which helped maintain his abstinence. However, he later began masturbating after having a “sudden urge.” He stated that when he is unable to engage in watching pornography or masturbation, he experiences a great deal of anxiety and other “withdrawal symptoms” such as penile hypersensitivity, gnawing of his teeth, and pain when his penis is not erect. Further history reported by the patient included a history of sexual trauma in childhood at seven years of age and spending hundreds of dollars in exchange for sexual acts upon himself and others.

Further workup included computed tomography of the brain, which revealed no acute intracranial pathology, as well as testosterone and prolactin levels, which were within normal limits. The patient was prescribed clonazepam 0.5 mg twice daily (BID) for anxiety with a plan to taper, mirtazapine 15 mg daily for depression and insomnia, paroxetine 30 mg daily for current depression, anxiety, and sexual compulsions, and risperidone 2 mg BID for mood stabilization. During his inpatient admission, the patient reported less sexual compulsions and noticeable improvement in mood overall. He continued to deny suicidal and homicidal ideations or any psychotic and manic symptoms. The patient was experiencing urges of sexual compulsion while in the hospital and requested a private room to avoid sexually harming a roommate. The patient was offered rehabilitation for sexual compulsions, but he declined. The patient was counseled on the importance of outpatient psychiatric follow-up and medication management, which he complied with. Upon discharge, the patient was sent back home to live with family in stable condition, and a follow-up appointment was arranged with an outpatient psychiatric clinic.

## Discussion

CBSD is defined by the following criteria in the ICD: engrossment in repetitive sexual activities to where the patient fails to meet obligations of life and is neglectful of responsibilities; failed reduction of sexual behavior on multiple occasions; continued sexual behavior despite negative consequences (i.e. loss of employment); or persistence of repetitive behavior even when little or no satisfaction is obtained [1]. These patients are also at a higher risk of comorbid substance use disorders, which can be seen in this particular patient as evidenced by his reliance on cannabis use to cope with the distress experienced from not being able to engage in sexual acts. Epidemiologically, it has been determined that males exhibit the disorder more than females, with 1-3% of adults affected, while the prevalence of hypersexual disorders within the general population among females is 3.1% [1,3]. The average age of onset is 18.7 years, but most patients do not seek treatment until approximately 37 years of age [3]. Our patient began to notice that his urges were causing him distress at the age of 27 and sought treatment at 31.

Despite increasing research on the psychological and neural mechanisms of CBSD, little is known about the efficacy of pharmacotherapy in people with this condition, though there is evidence that effective approaches exist in reducing sexual urges and distress in patients. Selective serotonin reuptake inhibitors (SSRIs) have been reported to control sexual obsessions and compulsions in CSBD by utilizing their sexual side effects such as difficulty with arousal [4]. A double-blind placebo-controlled random controlled trial demonstrated that paroxetine is safe and well tolerated in men with CSBD [5]. Based on clinical interviews, paroxetine was found to be more effective than placebo in reducing CSBD symptoms [5]. After one week of treatment with paroxetine, the patient reported feeling less distress with the absence of masturbation and experienced less sexual urges. Mood stabilizers, such as valproic acid and lithium, appear promising in the treatment of patients with bipolar disorder and compulsive sexual behaviors; however, this class of medications has an independent effect on reducing compulsive sexual behaviors in patients without comorbid bipolar disorder [6]. Topiramate, an antiepileptic that is used off-label as a mood stabilizer, can be considered in the treatment of CSBD as it was found to be effective in treating impulsivity in patients [7].

Naltrexone, an opioid receptor antagonist, has been previously used and found effective by reducing the symptoms in patients with CSBD [8]. Naltrexone additionally has a secondary mechanism of action by blocking the dopaminergic release in the nucleus accumbens to dampen the reward system of the brain [3]. Studies have shown that patients receiving polytherapy have reported a reduction in their sexual addiction, especially after multiple failed attempts with an SSRI. On the other hand, patients receiving monotherapy with naltrexone alone have noted a decline in their sexual urges and viewed pornography less [3]. In this patient, naltrexone was not used in trial as monotherapy for the management of his urges due to the unexpected benefit in reduction of his sexual compulsions by the risperidone that he was taking for bipolar disorder.

Psychotherapy techniques including cognitive behavioral therapy (CBT) in conjunction with pharmacotherapy were determined to be successful in managing patients with CSBD [9]. Acceptance and commitment therapy (ACT), a form of CBT, holds promise as a treatment for pornography addiction and CSBD. ACT focuses on the underlying processes of maladaptive behaviors in order to correct them [9]. Treatment with ACT resulted in an 85% reduction in viewing at-post treatment, with results being maintained at the three-month follow-up (83% reduction), and increases were also seen in measures of quality of life [9]. While the patient declined psychotherapy prior to discharge, he was advised to follow up with outpatient psychiatry to obtain a referral for psychotherapy when he felt ready.

## Conclusions

Though there is no FDA-approved treatment strategy currently for patients with concurrent CSBD and BPI, there are reports of medication trials that have been effective in treating these patients. SSRIs were reported to be effective against sexual compulsions, while mood stabilizers demonstrated the ability to depreciate the intensity of sexual impulsivity. On the other hand, some evidence has revealed that naltrexone monotherapy has shown reduction in sexual urges in males. In conjunction with any of these pharmacological treatment modalities, CBT should be utilized for optimal benefit to the patient.
